# A proposal of prior probability-oriented clustering in feature encoding strategies

**DOI:** 10.1371/journal.pone.0210146

**Published:** 2019-01-10

**Authors:** Yuki Shinomiya, Yukinobu Hoshino

**Affiliations:** School of System Engineering, Kochi University of Technology, Kami, Kochi, Japan; Liverpool John Moores University, UNITED KINGDOM

## Abstract

Codebook-based feature encodings are a standard framework for image recognition issues. A codebook is usually constructed by clusterings, such as the k-means and the Gaussian Mixture Model (GMM). A codebook size is an important factor to decide the trade-off between recognition performance and computational complexity and a traditional framework has the disadvantage to image recognition issues when a large codebook; the number of unique clusters becomes smaller than a designated codebook size because some clusters converge to close positions. This paper focusses on the disadvantage from a perspective of the distribution of prior probabilities and presents a clustering framework including two objectives that are alternated to the k-means and the GMM. Our approach is first evaluated with synthetic clustering datasets to analyze a difference to traditional clustering. In the experiment section, although our approach alternated to the k-means generates similar results to the k-means results, our approach is able to finely tune clusters for our objective. Our approach alternated to the GMM significantly improves our objective and constructs intuitively appropriate clusters, especially for huge and complicatedly distributed samples. In the experiment on image recognition issues, two state-of-the-art encodings, the Fisher Vector (FV) using the GMM and the Vector of Locally Aggregated Descriptors (VLAD) using the k-means, are evaluated with two publicly available image datasets, the Birds and the Butterflies. For the results of the VLAD with our approach, the recognition performances tend to be worse compared to the original VLAD results. On the other hand, the FV using our approach is able to improve the performance, especially in a larger codebook size.

## Introduction

Clustering is a fundamental technique for several purposes such as statistical analysis and data mining. The main purpose of clustering is to make groups called clusters. Each clustering technique has a specific objective to make groups, such as finding groups that minimize a quantization error and estimation of the appropriate distribution [1, 2]. This paper focusses on clustering in image recognition algorithms and presents an efficient objective.

In recent image recognition problems, a local feature framework is a key technique. This detects regions of interest on an image and describes a discriminative feature vector from each region [3–5]. The basic idea of codebook-based encodings is to capture the statistics of the distribution of local features extracted from an image. By treating local features as visual vocabularies appeared in an image, images can be processed in the same way as the natural language processing (NLP). In the NLP, specifically, the bag-of-words (BOW) model [6] expresses a document feature vector by assigning words existing in sentences to corresponding common words and counting their frequencies. For images, common visual words, called codebook, are constructed by clustering local features extracted from various images. The model in image recognition follows the same procedure as the BOW to represent image feature vectors. This approach is well-known as the bag-of-visual-words (BoVW) model [7], and its variants [8–13] have achieved excellent performance on several tasks, such as object recognition [8, 9, 11] and image retrieval [12, 13].

Gosselin et al. [10] have suggested that increasing the number of common visual vocabularies is an important factor for improving recognition performance. For instance, the best recognition rate has been observed with the largest vocabulary size in their experiment. It has also been reported that saturation of the recognition performances accompanying the increase the vocabulary size has not been observed. On the other hand, a huge vocabulary size becomes a cause of high computational complexity [10] and to possibly generate not suitable vocabularies due to the over-fitting to clustering samples [9]. Our previous study [14] has considered that the distribution of prior probability can be used to measure the quality of image feature vectors in codebook-based feature encoding strategies. In addition, optimization of the distribution does not require additional computational complexity in practical applications because it is an offline step in the image recognition pipeline.

This paper focuses on the codebook construction step and presents a clustering procedure, named prior probability oriented clustering, that generates a suitable codebook considered from the perspective of the distribution of prior probabilities [14] for feature encoding strategies. The contribution of this paper is threefold: first, our proposal has an explicit objective to optimize the codebook parameters. Second, it relaxes conditions to construct an optimized codebook, compared with the grid search used in [14]. Third, the framework uses general optimization techniques to minimize our objective.

The rest of this paper is organized as follows: the next section briefly reviews the relationship between clustering algorithms and feature encoding approaches; After that, we describe our proposal clustering framework; Then we analyze numerical characteristics of our proposal with synthetic clustering datasets; After that, we evaluate an effect for image recognition performance with image recognition datasets; Finally, we conclude this paper.

## Literature review of feature encodings

The basic pipeline for recognizing objects consists of the following steps.

1. *Extract local features*. A given image is first converted to a set of *d*-dimensional local features, $I={\left\{x_{i}\in R^{d}\right\}}_{i=1}^{N}$. The local features [3–5] have the robustness to some deformations, such as scale, rotation, occlusion.
2. *Encode to an image feature*. The above set is then encoded to a single feature vector based on a codebook, which is a set of basis vectors.
3. *Recognize object labels*. A discriminant model is used to predict object labels. Typically, the support vector machine (SVM) with a linear kernel is used because of its computational efficiency. The computational complexity at the model construction phase is a linear order with respect to the number of training samples [8, 15].

Here, the codebook is constructed in advance in an offline step. This section reviews the codebook construction step and the feature encoding step.

### Codebook construction

The basic clustering algorithms are the k-means [1] and the Gaussian mixture model (GMM) [2], which are usefull in several research fields [7, 16–18], such as image processing, signal processing, and physiology. The aim of the k-means algorithm is to find the clusters that minimize the quantization error between given samples and the corresponding mean vector. The mean vector is a representative position of a cluster, the quantization error is defined as a sum of square distances between a mean vector and the samples belonging to the cluster. The GMM constructs Gaussians that well represents the normal distribution of given samples. In general, clustering algorithms cannot directly find global optimal by any analysis. To find an suboptimal solution, the above algorithms follow an iterative procedure, called the expectation and maximization (EM) algorithm, for exploring local minima. This algorithm consists of the following two steps: the expectation step and the maximization step.

In the case of the k-means, let $X={\left\{x_{t}\in R^{d}\right\}}_{t=1}^{T}$ and $\Theta ={\left\{\mu_{k}\in R^{d}\right\}}_{k=1}^{K}$ respectively be the clustering samples and the model parameters, the objective function is defined as follows:

$$\begin{array}{c} J_{\text{k-means}}=\sum_{t=1}^{T}\sum_{k=1}^{K}p(x_{t};\mu_{k}){∥x_{t}-\mu_{k}∥}^{2}, \end{array}$$ (1)

where *J*_*k*−*means*_ is the objective value, which measures the quantization error between the samples and the clusters, *p*(*x*_*t*_; *μ*_*k*_) is a probability function that becomes 1 if *μ*_*k*_ is the nearest cluster to *x*_*t*_ and 0 otherwise, and ‖⋅‖ is the Euclidean norm operator. To minimize the quantization error, the k-means algorithm iteratively optimizes the model parameters with Eq (2) for the expectation step and Eq (3) for the maximization step.

$$\begin{array}{c} q_{t,k}=p(x_{t};\mu_{k}), \end{array}$$ (2)

$$\begin{array}{c} {\hat{\mu }}_{k}\leftarrow \frac{1}{T}\sum_{t=1}^{T}q_{t,k}(x_{t}-\mu_{k}). \end{array}$$ (3)

In the expectation step, the probabilities *q*_*t*,*k*_ of a sample *x*_*t*_ are computed using the current mean vectors. Then, the maximization step updates the positions. The EM algorithm iterates the above two steps until termination criteria, such as a designated maximum number of iterations and the convergence of the moves, are satisfied.

Fitting the GMM model also uses the EM algorithm. The GMM model contains ${\left\{w_{k}\in R,\mu_{k}\in R^{d},\Sigma_{k}\in R^{d\times d}\right\}}_{k=1}^{K}$, where *μ*_*k*_ and *Σ*_*k*_ denote the mean and the covariance matrix of the *k*-th Gaussian and *w*_*k*_ is a mixing weight for mixing *K* Gaussians. The mixing weight *w*_*k*_ is also called “prior probability”, which means the ease of assignment to the *k*-th Gaussian.

$$q_{t,k}=\overset{K}{\underset{k=1}{\sum }}w_{k}p\left(x_{t};\mu_{k},\Sigma_{k}\right),$$ (4)

$$p\left(x_{t};\mu_{k},\Sigma_{k}\right)=\frac{1}{\sqrt{2\pi^{d}\left|\Sigma_{k}\right|}}\text{exp}\left(-\frac{1}{2}\left(x_{t}-\mu_{k}\right)\Sigma_{k}^{-1}{\left(x_{t}-\mu_{k}\right)}^{⊤}\right),$$ (5)

$$\begin{array}{c} {\hat{\mu }}_{k}\leftarrow \frac{\sum_{t=1}^{T}q_{t,k}x_{t}}{\sum_{t=1}^{T}q_{t,k}},\;{\hat{\Sigma }}_{k}\leftarrow \frac{\sum_{t=1}^{T}q_{t,k}\left(x_{t}-{\hat{\mu }}_{k}\right){\left(x_{t}-{\hat{\mu }}_{k}\right)}^{⊤}}{\sum_{t=1}^{T}q_{t,k}},\;{\hat{w}}_{k}\leftarrow \frac{\sum_{t=1}^{T}q_{t,k}}{\sum_{t=1}^{T}\sum_{k=1}^{K}q_{t,k}}, \end{array}$$ (6)

### Feature encoding

As introduced in the previous section, the BoVW is the simplest approach to represent image features and well performs in image recognition applications. The BoVW usually uses the k-means codebook. Let $I={\left\{x_{i}\in R^{d}\right\}}_{i=1}^{N}$ be a set of *d*-dimensional local descriptors extracted from an image, the BoVW feature is defined as:

$$\begin{array}{c} F_{\text{BoVW}}={\left[f_{1},\cdots ,f_{k},\cdots ,f_{K}\right]}^{⊤},\;f_{k}=\sum_{i=1}^{N}p\left(x_{i};\mu_{k}\right), \end{array}$$ (7)

where $f_{k}\in R^{1}$ is the frequency of the local descriptors assigned to the *k*-th visual vocabulary. For precisely capture image information, the BoVW requires a huge codebook, because the dimensionality of the BoVW is equal to a codebook size *K*, and it increases the computational cost, such as the finding nearest neighbors as in Eq (2). Recently developed approaches [8, 13] relax this issue by capturing higher order statistics on *d*-dimensional local feature space with a smaller codebook. In recent reports, the Fisher Vector (FV) [8, 9] and the Vector of Locally Aggregated Descriptors (VLAD) [12, 13] encodings are well known as state-of-the-art approaches.

The FV supplements two higher-order statistics with the GMM codebook, in addition to the frequency as follows:

$$\begin{array}{c} F_{\text{FV}}=[F_{1}^{\left(w\right)},\cdots ,F_{k}^{\left(w\right)},\cdots ,F_{K}^{\left(w\right)},F_{1}^{\left(\mu \right)},\cdots ,F_{k}^{\left(\mu \right)},\cdots ,F_{K}^{\left(\mu \right)},F_{1}^{\left(\sigma \right)},\cdots ,F_{k}^{\left(\sigma \right)},\cdots ,F_{K}^{\left(\sigma \right)}], \end{array}$$ (8)

where $F^{\left(w\right)}\in R^{1}$, $F^{\left(\mu \right)}\in R^{d}$, and $F^{\left(\sigma \right)}\in R^{d}$ respectively denote frequency, mean, and covariance. These are captured as:

$$F_{k}^{\left(w\right)}=\frac{1}{N\sqrt{w_{k}}}\sum_{i=1}^{N}\left(q_{i,k}-w_{k}\right),$$ (9)

$$F_{k}^{\left(\mu \right)}=\frac{1}{N\sqrt{w_{k}}}\sum_{i=1}^{N}q_{i,k}\frac{x_{i}-\mu_{k}}{\sigma_{k}},$$ (10)

$$F_{k}^{\left(\sigma \right)}=\frac{1}{N\sqrt{2w_{k}}}\sum_{i=1}^{N}q_{i,k}\left[\left(\frac{x_{i}-\mu_{k}}{\sigma_{k}}\right)-1\right],$$ (11)

where the Gaussians are assumed to have diagonal covariances because of the derivation [8] and computational reasons [9, 10]. Therefore, a FV signature have *K*(2*d* + 1)-dimensions. The VLAD captures only mean statistics by aggregating the residuals between the local features and the mean vectors of the codebook as follows:

$$F_{\text{VLAD}}=\left[F_{1}^{\left(\mu \right)},\cdots ,F_{k}^{\left(\mu \right)},\cdots ,F_{K}^{\left(\mu \right)}\right],$$ (12)

$$F_{k}^{\left(\mu \right)}=\overset{N}{\underset{i=1}{\sum }}q_{i,k}\left(x_{i}-\mu_{k}\right),$$ (13)

where the dimensionality of a VLAD signature is *Kd*.

## The prior probability-oriented clustering

As described in the above section, the distribution or prior probabilities *w*_*k*_ is an important factor to measure the quality of the feature encodings. The aim of the prior probability-oriented clustering is to mainly minimize the variance of prior probabilities.

The k-means and the GMM follow the iterative procedures because there is no analytic solution for unknown samples [19], as described in the literature reviews. Even in our approach, the procedure uses general optimization algorithms for finding local minima. The objective function is defined as the following equation and consists of two terms:

$$\begin{array}{c} J=\underset{\text{main}\;\text{objective}\;\text{term}}{\underset{︸}{\sum_{k=1}^{K}\left|w_{k}-\bar{w}\right|}}+\lambda \underset{\text{regularization}\;\text{term}}{\underset{︸}{\frac{1}{T}\sum_{t=1}^{T}d{\left(x_{t};\Theta \right)}^{2}}}. \end{array}$$ (14)

The main objective term is an approximated measure of the variance calculation $\frac{1}{K}\sum_{k=1}^{K}{\left(w_{k}-\bar{w}\right)}^{2}$, where $\bar{w}$ is the average of the prior probabilities. *d*(*x*_*t*_;Θ) is a regularizer that measures the quantization error between the *t*-th sample and its nearest cluster mean. It serves to smooth solution space. For example, when clustering a number of samples with only the main objective, the solution space might be discrete, which means that small changes of candidate mean positions probably give the same objective value. λ is a weighting factor that controls which the main objective term and the regularization term is relatively more important. In our concept, λ is set to a small value to emphasize the main objective. An effect of λ is discussed in the next section.

As an optimization framework, a black-box optimization framework is used to minimize our objective shown in Eq (14), which does not require any constraints, such as derivation, for objective functions. In the next section, some black-box optimization frameworks are evaluated with synthetic clustering datasets. The general optimization procedure to find suboptimal solution is as follows:

1. generate initial mean vectors by k-means++ algorithm [20];
2. repeat:
3. evaluate the our proposal objective function as in Eq (14), where the detail on how to evaluate the regularization term is described in below subsections;
4. update mean vectors by a black-box optimization framework;
5. until the number of iterations reaches.

### Hard clustering alternated to the k-means

In this case, the clustering problem is defined as minimizing the variance of prior probabilities while minimizing the quantization error. The quantization error is defined as follows:

$$\begin{array}{c} d\left(x_{t};\Theta \right)=\sum_{k=1}^{K}q_{t,k}∥x_{t}-\mu_{k}∥, \end{array}$$ (15)

The procedure of Eq (15) is as follows.

1. predict assignment probabilities *q*_*t*,*k*_ for all clustering samples $X={\left\{x_{t}\right\}}_{t=1}^{T}$, using Eq (2);
2. compute prior probabilities, in the same manner as the GMM, as: $w_{k}=\frac{1}{T}\sum_{t=1}^{T}q_{t,k}$;
3. evaluate the objective value, using Eq (14) with Eq (15).

### Soft clustering alternated to the GMM

In order to estimate Gaussians with only mean vectors, each posterior probability of a sample for the *k*-th cluster is approximated with the nearest search as in Eq (2) as:

$$\begin{array}{c} q_{t,k}=p(x_{t};\mu_{k}), \end{array}$$ (16)

It is a natural approximation because of the following reasons.

- Many GMM implementations [21, 22] use the k-means initialization before the EM iterations.
- The distribution of posterior probabilities is peaky in general, a posterior probability closes to 1 and others become 0.
- The term of the Mahalanobis distance is dominant to predict the posterior probability function in Eq (4).

The regularization term is calculated in the same way as the distance metric, the Mahalanobis distance, of the GMM as follows:

$$\begin{array}{c} d\left(x_{t};\Theta \right)=\sum_{k=1}^{K}q_{t,k}{\left(x_{t}-\mu_{k}\right)}^{⊤}\Sigma_{k}^{-1}\left(x_{t}-\mu_{k}\right), \end{array}$$ (17)

The procedure of the soft objective is as follows.

1. predict assignment probabilities *q*_*t*,*k*_ for all clustering samples $X={\left\{x_{t}\right\}}_{t=1}^{T}$, using Eq (2);
2. estimate *w*_*k*_ and *Σ*_*k*_ in the same manner as Eq (6);
3. evaluate the objective value, using Eq (14) with Eq (17).

## Numerical analysis

In this section, we first explore which optimization framework is better for our objective function. Then, we analyze the characteristics of the traditional clustering approaches, described in the previous section, and our proposal clustering approach. To evaluate these algorithms, we used two synthetic clustering datasets: the A-sets [23] and the S-sets [24], which are publicly available [25]. The A-sets and the S-sets respectively consist of A1, A2, and A3 for varying the number of clusters and S1, S2, and S3 for varying spatial complexity [23, 24]. Their statistics are shown in Table 1.

**Table 1:** Statistics of the A-sets and the S-sets.

	# of samples	# of clusters
A-sets (A1)	3,000	20
A-sets (A2)	5,250	35
A-sets (A3)	7,500	50
S-sets (S1)	5,000	15
S-sets (S2)	5,000	15
S-sets (S3)	5,000	15

The following shows the experimental setup.

- *Parameters in the k-means and the GMM*. The initial algorithm was the k-means++ algorithm [20], which improves the stability of solutions. The covariance matrices of Gaussians were assumed to diagonal. For the analysis, the implementations of the scikit-learn package [26] with the Python programming language were used. The termination criterion was that the number of iterations of the EM procedure reaches 2,000 times.
- *Parameters in our proposal*. As optimization frameworks, the Nelder-Mead (NM) [27], the Subplex [28], the Constrained BY Linear Approximation (COBYLA) [29], the NEWUOA [30], and the AUGmented LAGrangian algorithm (AUGLAG) [31, 32], which have been implemented in the NLOPT library [33], were evaluated. These algorithms are usually used for problems whose solution space structure is unknown and do not require any additional information, such as derivative of solution space, other than objective function. The initial position was set to the concatenated mean vectors generated by the k-means algorithm with 10 iterations. Therefore, the optimization frameworks explore the *Kd*-dimensional space. The termination criterion was that the number of the evaluations of the objective function reaches 2,000 times. The weighting factor was set to λ = 10^−9^.
- *Clustering samples*. The subsets, A1, A2, and A3, of the A-sets, were used for hard clustering, and the subsets, S1, S2, and S3, of the S-sets were used for soft clustering. The samples of each subset were linearly normalized that the values in each dimension fit within the range of [0, 1].

### Comparison of the optimization algorithms

Tables 2 and 3 show the objective values optimized by the optimization algorithms with the weighting factor λ = 10^−9^, where each value shows the best value over five trials and the values for the baselines were obtained only by the main objective term in Eq (14).

**Table 2:** Comparison of the optimized objective values regarding the optimization algorithms on the A-sets. The boldface indicates the best objective values in each subset.

Solver	k-means	NM	SBPLX	COBYLA	NEWUOA	AUGLAG
A1	0.0167	0.0020	**0.0007**	0.0040	0.0060	0.0040
A2	0.0114	0.0038	**0.0015**	0.0038	0.0038	0.0042
A3	0.0396	0.0045	**0.0019**	0.0029	0.0053	0.0037

**Table 3:** Comparison of the optimized objective values regarding the optimization algorithms on the S-sets. The boldface indicates the best objective values in each subset.

Solver	GMM	NM	SBPLX	COBYLA	NEWUOA	AUGLAG
S1	0.5424	0.0220	0.0107	**0.0105**	0.0276	0.0131
S2	0.5636	0.0185	**0.0089**	0.0104	0.0217	0.0107
S3	0.4632	0.0088	**0.0016**	0.0104	0.0031	0.0092

For all the optimization algorithms, the optimized values were smaller than the baseline results. Specifically, the Subplex gave the smallest objective values on all datasets except for S1. For the Subplex results on the A-sets, the objective values increased as the number of samples or clusters increases, in order to A1, A2, and A3. The mean value of prior probabilities is always 1/K because of the probabilistic constraint, and the large cluster size is expected to a cause to decrease the value of our main objective term. Therefore, our proposal with the hard objective might not effective for large samples or cluster size. For the S-sets, the results of soft objective suggest an advantage to the spatial complexity of sample distribution, the objective value decrease as sample distribution is more complicated, in all the optimization algorithms. In the results on S3, the Subplex showed especially better value compared with the results of the other optimization algorithms.

### Qualitative comparison of the constructed clusters

**Fig 1:**
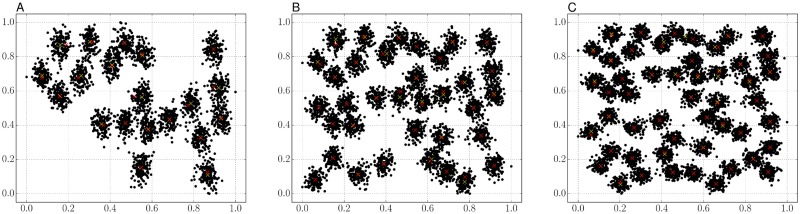
Comparison of the generated mean vectors on the A-sets. (A–C) The positions of the mean vectors generated by the k-means (yellow crosses) and our proposal (red crosses) for A1, A2, and A3 respectively.

Fig 1 shows the estimated mean vectors on the A-sets. Many positions of ours, indicated by the red crosses in Fig 1(A)–1(C), were close to the positions of the k-means, indicated by the yellow crosses. The k-means gave similar results to ours in the A-sets, while the k-means objective shown in Eq (1) does not have a term to minimize the variance of prior probabilities. Therefore, our proposal finely tunes mean positions for the main objective in Eq (14).

**Fig 2:**
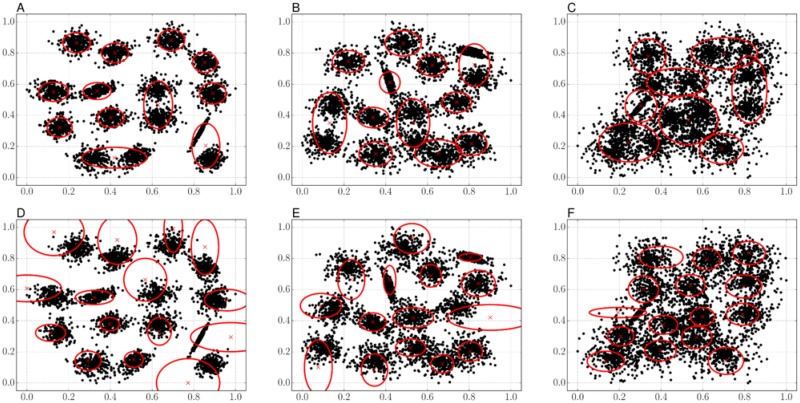
Illustrative examples of the estimated Gaussians on the S-sets. (A–C) The estimated Gaussians by the GMM for S1, S2, and S3 respectively. (D–F) The estimated Gaussians by our proposal for S1, S2, and S3 respectively. The black circles show the clustering sample positions, and the red cross and the ellipse respectively show the mean position and the confidence corresponding each Gaussian.

Fig 2 shows the estimated Gaussians on the S-sets. The GMM generated fewer Gaussians than the designated number of clusters, as in Fig 2(A)–2(C); three Gaussians for S1 and S2, and seven Gaussians for S3 were converged to the same positions of other Gaussians. It is considered that the number of Gaussians becomes smaller as clustering samples are more complicated. In the codebook construction step, lots of local features, usually 100K–1M, are used as clustering samples. Therefore, this characteristic has a disadvantage, that the number of unique visual-words becomes less than a designated codebook size when generating a codebook. Specifically, some components of an image signature have the same trend due to the overlapping of Gaussians or become always zeros when using the approximation of assignment probability, as in Eq (16). On the other hand, the results of ours in Fig 2(D)–2(F) show the fully distributed 15 Gaussians for the clustering samples. For spatially complicated samples as in Fig 2(F), the Gaussians were properly fitted to the sample distribution, intuitively. However, some Gaussians might not properly express for the sparsely scattered samples such as Fig 2(D) and 2(E). This characteristic is matched with the results of the comparison of the optimization algorithms, shown in Table 3; the objective value becomes better as the samples have more spatial complexity in our proposal. In addition, it suggests that our proposal possibly better for the codebook construction.

### Effect of the weighting factor

**Fig 3:**
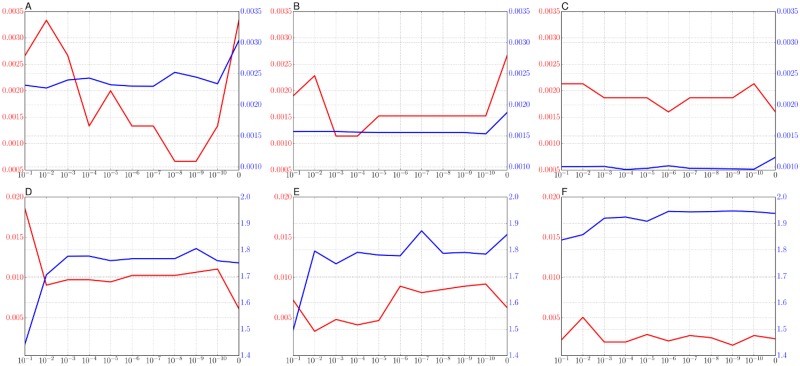
The trends of the optimized objective values with respect to the weighting factor. (A-C) The trends of the hard objective on A1, A2, and A3 of the A-sets. (D-F) The trends of the soft objective on S1, S2, and S3 of the S-sets. The horizontal axis shows the weighting factor λ and the vertical axis shows the main objective term and the regularization term. The red line shows the main objective values and the blue line shows the regularization value without weighting by λ shown in Eq (14).

Fig 3 shows the trends of objective values with respect to the weighting factor λ on the A-sets and the S-sets.

The ranges of the main objective term and the regularization term are in [0, 0.0035] and [0.001, 0.003] for the A-sets, and in [0, 0.02] and [1.4, 2] for the S-sets. The minimum of the main term ideally becomes 0 when all prior probabilities *w_k_* are the same value 1/K. The regularization term never becomes 0 because a cluster consists of scattered samples. For the results on the A-sets in Fig 3(A)–3(C), the values of the regularization term decrease as the number of clusters increase because the dispersion of samples in each cluster is small in order to A1, A2, and A3 in Fig 1. For the S-sets in Fig 3(D)–3(F), the values of the regularization term increase in order to S1, S2, and S3 because of the increase of the spatial complexity.

As shown in Fig 3(A) and 3(B), a larger weighting factor probably is a cause to increase the main objective term, where the objective value needs to be smaller. We consider that a relatively smaller weighting factor (λ < 10^−7^) correctly works, especially in Fig 3(A). On the whole trends in Fig 3(A)–3(F), there was no clear trend of the main objective regarding the weighting factor. The tendency to the regularization term is relatively intuitive, in particular for the soft objective, the quantization error decreases as the weighting coefficient increases.

## Experiments with image databases

This section evaluates our proposal on image recognition tasks with the following image datasets: *Birds* [34] and *Butterflies* [35] provided by Ponce Group.

The Birds dataset consists of 600 images categorized into six bird species, where each category has 100 images. The Butterflies dataset has 619 images of seven different butterflies. Each category has about 40 to 130 images. The above two datasets are composed of visually similar images.

In the experiments with the above datasets, we used the same parameter setup except for numbers of training images to construct a codebook and a discriminant model.

We used SURF [5] as the local feature framework. To extract SURF features, we followed the dense sampling strategy [36], which SURF features were described from the intersection points of the lattice of six pixels intervals, with multiple scale regions, 16, 20, 24, and 28 pixels for each point, where each image was resized so that the long side was 300 pixels. Each SURF feature was projected to 8-dimensional space by the Principle Component Analysis before constructing a codebook and encoding an image feature [37].

To construct a codebook, clustering samples were the SURF features extracted from 10 images from each category for the Birds and 5 images from each category for the Butterflies, where we decided about 10% of the smallest number of images of their categories. The codebook sizes of the five different patterns *K* = {16, 32, 64, 128, 256} were used. The termination criterion for the k-means and the GMM was set to 30 iterations because they do not converge sometimes. For our proposal, the termination criterion was set to 2,000 evaluations of the objective function. Gaussians of the GMM and our proposal with soft objective were assumed to diagonal covariance. The weighting factor of our objective was set to λ = 10^−9^. The k-means and ours with hard objective were used for the VLAD encoding, and the GMM and ours with soft objective were used for the FV encoding. Here, the dimensionality of image signatures depends on an experimental setting, for example, the number *K* of clusters and the number *d* of the dimension of local features. As introduced in the literature review section, the dimensionality becomes *Kd* for the VLAD and *K*(2*d* + 1) for the FV. Furthermore, the VLAD and the FV have 2, 048 and 4, 352 features when *K* = 32 and *d* = 8.

The SVM with the linear kernel, implemented in [26], was used as a discriminant model. The number of training images for each category was {30, 40, 50} for the Birds and {20, 30, 40} for the Butterflies. The training images were randomly selected, and the rest images were used for the test. The recognition accuracy was the ratio of the number of correctly recognized images for the number of test images. We measured by the average over five different training and test images.

**Fig 4:**
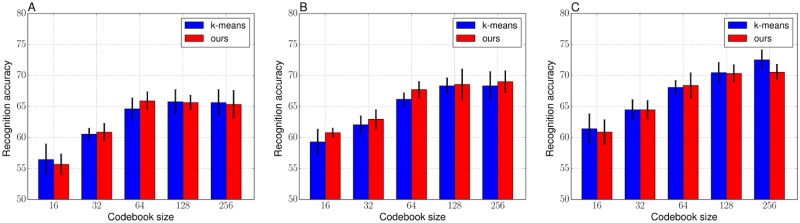
Recognition accuracies of the VLADs with the k-means and ours (hard objective) codebooks on the Birds. (A) 30 images per category for training. (B) 40 images per category for training. (C) 50 images per category for training.

**Fig 5:**
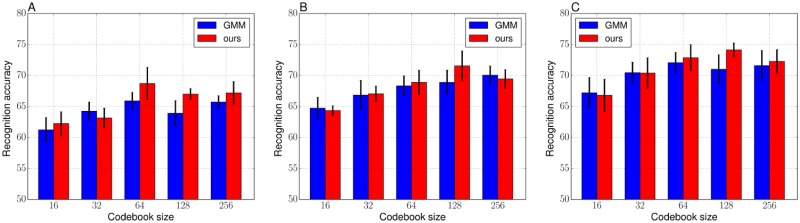
Recognition accuracies of the FVs with the GMM and ours (soft objective) codebooks on the Birds. (A) 30 images per category for training. (B) 40 images per category for training. (C) 50 images per category for training.

Figs 4 and 5 respectively show the average recognition accuracies of the VLAD and the FV on the Birds dataset. Tables 4 and 5 show the detailed values (mean accuracy and standard deviation over the five trials) corresponding to Figs 4 and 5. For the results of Fig 4, the baseline, the VLAD with the k-means codebook, and the VLAD with our hard objective showed similar performances regardless of the parameters such as the number of training images and the codebook sizes. As discussed in the numerical analysis section, the hard objective mainly performs to finely tune mean positions, the k-means and our hard objective clustering potentially construct similar codebooks. Table 6 shows the objective values of the codebooks used in Fig 4. When the codebook size is not greater than 64, the hard objective showed significantly better objectives compared with the k-means objectives. However, when the codebook size is greater than or equal to 64, they showed almost the same objectives. The k-means is possible to construct suitable clusters from the perspective of the variance of prior probabilities, regardless of the size of the clustering sample set or the codebook size, as shown in Fig 4. The hard objective might have difficulty to effectively optimize codebook for large clustering sample set or large codebook sizes, as discussed in the qualitative comparison in the numerical section. On the other hand, the FV with our soft objective often showed better performances compared with the FV with the GMM codebook, especially when the codebook size is 128. When the codebook size was small, *K* = 16 and *K* = 32, there is no significant difference of the recognition performances of the baseline and the FV with the soft objective. For the larger codebook size, the FV with the soft objective performed better accuracies. Moreover, our soft objective with a relatively larger codebook size was more effective for the case that training image set is smaller compared with the test image set. The highest mean recognition accuracy was achieved when the codebook size was 64, 128, and 128 respectively for 30, 40, and 50 training images per category. Therefore, an increase in the codebook size does not necessarily lead to improving recognition performance, the codebook size *K* = 64 or *K* = 128 might be enough for the Birds dataset. Table 7 shows the objective values of the codebooks used in Fig 5. In contrast to the trend of the objective values of the hard objective, the soft objective could maintain the better values, shown in Table 7, even when the codebook size is increased. As with the discussions in numerical analysis, the soft objective is able to construct a suitable codebook, from the perspective of the variance of prior probability, even in image recognition tasks. When comprehensively comparing the results of the VLADs in Table 4 and the FVs in Table 5, the FV with our soft objective (*K* = 64) showed the best accuracy of 68.71 when the training images were 30 for each category. The FV with ours (*K* = 128) also showed the best accuracies as follows: 71.56 for 40 training images and 74.13 for 50 training images.

**Table 4:** Recognition performance (mean accuracy ± standard deviation) of the VLADs with the k-means and ours (hard objective) codebooks on the Birds, corresponding to the Fig 4.

	Codebook size *K*				
Method	16	32	64	128	256
30 images from each category, corresponding to Fig 4(A)					
k-means	56.43 ± 2.52	60.52 ± 0.98	64.62 ± 1.76	65.76 ± 1.97	65.62 ± 2.11
ours	55.67 ± 1.69	60.86 ± 1.43	65.90 ± 1.47	65.62 ± 1.19	65.33 ± 2.26
40 images from each category, corresponding to Fig 4(B)					
k-means	59.28 ± 2.08	62.06 1.44	66.17 ± 1.05	68.33 ± 1.30	68.33 ± 2.30
ours	60.78 ± 0.73	62.94 ± 1.55	67.72 ± 1.33	68.56 ± 2.49	69.00 ± 1.77
50 images from each category, corresponding to Fig 4(C)					
k-means	61.40 ± 2.43	64.47 ± 1.64	68.07 ± 1.14	70.47 ± 1.65	72.53 ± 1.63
ours	60.87 ± 2.01	64.47 ± 1.50	68.40 ± 2.05	70.33 ± 1.41	70.53 ± 1.29

**Table 5:** Recognition performance (mean accuracy ± standard deviation) of the FVs with the GMM and ours (soft objective) codebooks on the Birds, corresponding to the Fig 5.

	Codebook size *K*				
Method	16	32	64	128	256
30 images from each category, corresponding to Fig 5(A)					
GMM	61.24 ± 1.99	64.24 ± 1.50	65.90 ± 1.39	63.90 ± 2.05	65.71 ± 1.03
ours	62.24 ± 1.92	63.14 ± 1.62	68.71 ± 2.63	67.00 ± 0.91	67.19 ± 1.85
40 images from each category, corresponding to Fig 5(B)					
GMM	64.72 ± 1.77	66.83 ± 2.41	68.33 ± 1.64	68.89 ± 2.02	70.06 ± 1.51
ours	64.33 ± 0.80	67.06 ± 1.26	68.89 ± 1.98	71.56 ± 2.43	69.44 ± 1.50
50 images from each category, corresponding to Fig 5(C)					
GMM	67.20 ± 2.50	70.47 ± 1.71	72.07 ± 1.68	71.00 ± 2.37	71.60 ± 2.44
ours	66.80 ± 2.60	70.40 ± 2.48	72.87 ± 2.14	74.13 ± 1.13	72.27 ± 1.94

**Table 6:** The objective values of the k-means and ours with the hard objective with respect to the codebook size on the Birds.

	Codebook size				
Method	16	32	64	128	256
k-means	0.2210	0.1940	0.1923	0.1995	0.2082
ours (hard)	0.0071	0.0205	0.0895	0.1759	0.2126

**Table 7:** The objective values of the GMM and ours with the soft objective with respect to the codebook size on the Birds.

	Codebook size				
Method	16	32	64	128	256
GMM	0.4139	0.3248	0.2773	0.3240	0.3171
ours (soft)	0.0014	0.0297	0.0580	0.1268	0.1508

**Fig 6:**
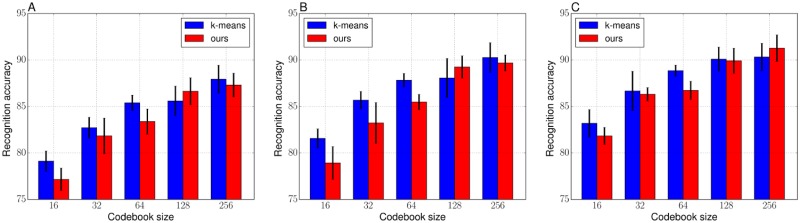
Recognition accuracies of the VLADs with the k-means and ours (hard objective) codebooks on the Butterflies. (A) 20 images per category for training. (B) 30 images per category for training. (C) 40 images per category for training.

**Fig 7:**
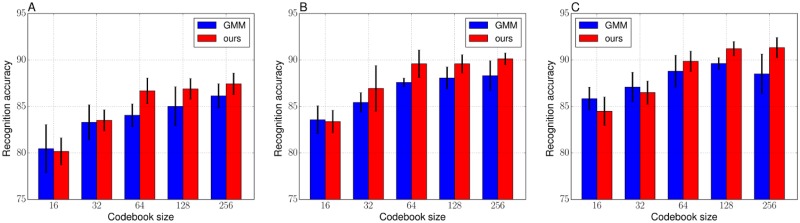
Recognition accuracies of the FVs with the GMM and ours (soft objective) codebooks on the Butterflies. (A) 20 images per category for training. (B) 30 images per category for training. (C) 40 images per category for training.

Figs 6 and 7 respectively show the average recognition accuracies of the VLAD and the FV on the Butterflies dataset. Tables 8 and 9 show the detailed values (mean accuracy and standard deviation over the five trials) corresponding to Figs 6 and 7. From the results in Fig 6, the hard objective may deteriorate recognition performance when codebook size is smaller than or equal to 64. In addition, the objective values of the hard objective, shown in Table 10, were not enough optimized as with the case of the Birds dataset. For the results with the FV, the GMM and the soft objective showed similar performances when the codebook size is small. As with the numerical analysis, a smaller codebook size has less influence on the convergence of the Gaussians, and the GMM makes it easier to converge Gaussians to the same positions when the clustering samples is spatially complicatedly distributed and a codebook size is large. However, it improved recognition performances clearly when the codebook size is larger than 32, in all of the training images per category and lead to improve recognition performances when the codebook size was 256. Table 11 shows the objective values of the soft objective with respect to the number of codebook size and these values suggest that our framework is able to estimate proper Gaussians regardless of the codebook size. In the case of comparing the results of the VLADs in Table 8 and the FVs in Table 9, the VLAD with the k-means (*K* = 256) showed best accuracy: 87.93 for 20 training images and 90.27 for 30 training images. On the other hand, for the 40 training images, the FV with ours (*K* = 256) showed the best accuracy of 91.33.

**Table 8:** Recognition performance (mean accuracy ± standard deviation) of the VLADs with the k-means and ours (hard objective) codebooks on the Butterflies, corresponding to the Fig 6.

	Codebook size *K*				
Method	16	32	64	128	256
20 images from each category corresponding to Fig 6(A)					
k-means	79.12 ± 1.10	82.71 ± 1.13	85.39 ± 0.84	85.59 ± 1.60	87.93 ± 1.51
ours	77.16 ± 1.21	81.84 ± 1.91	83.38 ± 1.36	86.64 ± 1.45	87.31 ± 1.28
30 images from each category corresponding to Fig 6(B)					
k-means	81.56 ± 1.02	85.67 ± 0.95	87.82 ± 0.73	88.07 ± 2.09	90.27 ± 1.59
ours	78.92 ± 1.76	83.23 ± 2.20	85.48 ± 0.83	89.24 ± 1.20	89.68 ± 0.87
40 images from each category, corresponding to Fig 6(C)					
k-means	83.19 ± 1.48	86.67 ± 2.11	88.85 ± 0.60	90.09 ± 1.30	90.32 ± 1.47
ours	81.83 ± 0.93	86.31 ± 0.71	86.73 ± 0.99	89.91 ± 1.35	91.27 ± 1.43

**Table 9:** Recognition performance (mean accuracy ± standard deviation) of the FVs with the GMM and ours (soft objective) codebooks on the Butterflies, corresponding to the Fig 7.

	Codebook size *K*				
Method	16	32	64	128	256
20 images from each category corresponding to Fig 7(A)					
GMM	80.46 ± 2.59	83.30 ± 1.88	84.05 ± 1.23	85.01 ± 2.11	86.14 ± 1.30
ours	80.17 ± 1.46	83.51 ± 1.13	86.68 ± 1.38	86.89 ± 1.12	87.43 ± 1.16
30 images from each category corresponding to Fig 7(B)					
GMM	83.57 ± 1.50	85.43 ± 1.06	87.58 ± 0.47	88.07 ± 1.18	88.31 ± 1.60
ours	83.37 ± 1.21	86.94 ± 2.45	89.58 ± 1.49	89.58 ± 0.99	90.12 ± 0.63
40 images from each category, corresponding to Fig 7(C)					
GMM	85.84 ± 1.24	87.08 ± 1.60	88.79 ± 1.72	89.62 ± 0.63	88.50 ± 2.14
ours	84.48 ± 1.53	86.49 ± 1.24	89.85 ± 1.10	91.21 ± 0.78	91.33 ± 1.08

**Table 10:** The objective values of the k-means and ours with the hard objective with respect to the codebook size on the Butterflies.

	Codebook size				
Method	16	32	64	128	256
kmeans	0.1962	0.1580	0.1966	0.1828	0.2106
ours (hard)	0.0011	0.0201	0.1030	0.1915	0.1883

**Table 11:** The objective values of the GMM and ours with the soft objective with respect to the codebook size on the Butterflies.

	Codebook size				
Method	16	32	64	128	256
GMM	0.3810	0.3322	0.3407	0.2981	0.2961
ours (soft)	0.0018	0.0191	0.0557	0.1017	0.1352

## Conclusions

This paper focussed on clustering from the perspective of the variance prior probabilities and presented the clustering frameworks, namely hard and soft objectives, that are respectively alternative to basic approaches such as the k-means and the GMM. In the numerical analysis, four optimization frameworks were evaluated with synthetic clustering datasets. The results of all of the frameworks were better than the basic clusterings. Especially, it showed that the Subplex optimizer is able to give better objective values from the perspective of the variance of prior probabilities and to construct intuitively appropriate clusters for complicatedly distributed clustering samples. In the experiment with image datasets, the hard objective was probably not effective for the VLAD encoding because the objective values became worse compared with the k-means results as the number of clusters increase. On the other hand, the FV encoding with the soft objective showed improvements in recognition performance regardless of some parameters such as the codebook size and the ratio of training and test images.
